# Redox Priming Ameliorates Salinity Tolerance of Seeds and Seedlings of the Coastal Halophyte Grass *Urochondra setulosa*

**DOI:** 10.3390/plants15030350

**Published:** 2026-01-23

**Authors:** Sadiq Hussain, Farah Nisar, Sahar Abbas, Abdul Hameed, Brent L. Nielsen

**Affiliations:** 1 Dr. Muhammad Ajmal Khan Institute of Sustainable Halophyte Utilization, University of Karachi, Karachi 75270, Pakistan; shussain.ku@gmail.com (S.H.); farah.nisar@aku.edu (F.N.); raghib72@gmail.com (S.A.); 2 Faculty of Arts and Sciences, Aga Khan University, Karachi 74800, Pakistan; 3 Department of Microbiology and Molecular Biology, Brigham Young University, Provo, UT 84602, USA

**Keywords:** salinity tolerance, seed germination, seed priming, redox priming, halophyte grasses, seedling growth, fodder crop

## Abstract

Low salinity tolerance during germination and early seedling establishment limits large-scale cultivation of halophytes for forage, food, restoration, and conservation purposes. This study evaluates the potential of redox priming to enhance salt tolerance in the perennial C_4_ halophyte grass *Urochondra setulosa*, which could be used as a revegetation and phytoremediation crop for coastal saline lands. Fresh seeds were found to be non-dormant with ~90% mean final germination (MFG) in distilled water. Redox priming, including hydrogen peroxide (H_2_O_2_), melatonin (MT), sodium nitroprusside (SNP; a nitric oxide donor), and ascorbic acid (AsA), significantly accelerated the germination rate index (GRI) and reduced mean germination time (MGT) without altering MFG under non-saline conditions. Salinity severely suppressed germination, as unprimed seeds reached only ~1% MFG with ~99% germination reduction (GR) and near-zero germination stress tolerance index (GSTI) at 200 mM NaCl. All priming treatments significantly improved MFG, GRI, and GSTI and decreased GR, with H_2_O_2_ priming showing the highest amelioration. Ungerminated seeds from all treatments recovered ~90% germination capacity in water, indicating enforced dormancy owing to osmotic constraints. Salinity did not impair growth in unprimed seedlings. However, MT priming uniquely enhanced total length, leaf area, and seedling vigor index (SVI) at 200 mM NaCl, while MT and SNP priming resulted in the highest chlorophyll and carotenoid contents. Multivariate analyses confirmed MT’s consistent superiority across traits under stress. Thus, H_2_O_2_ priming optimizes germination, while MT priming improves seedling vigor and offers a practical, targeted strategy to improve early-stage salinity tolerance in *U. setulosa* for coastal revegetation and sustainable saline agriculture.

## 1. Introduction

The world population is increasing at an alarming rate and may cross 9 billion by 2050 [1,2]. As a result, twice the agricultural productivity of today will be required to ensure food security in the future [1,3]. About 690 million people are already undernourished worldwide, and this number will exceed 840 million by 2030 [4]. In contrast, degradation of agricultural lands due to anthropogenic and global climate change-driven salinization is exerting immense pressure on conventional agriculture, as most crops are sensitive to salinity [4,5,6]. Moreover, success in developing salinity-tolerant varieties of crops has been marginal [7]. Hence, cultivation of naturally tolerant halophytes such as coastal C_4_ grasses on barren saline/arid lands with salty water irrigation could be a sustainable alternative to supplement food, fodder, fuel, and medicinal requirements of the fast-growing human population [8]. However, a crop candidate suitable for one area may not be suitable for other regions; therefore, there is a dire need to explore halophytic crop candidates from the local flora.

*Urochondra setulosa* (Trin.) C.E. Hubbard (Poaceae) is the only species in the genus *Urochondra* and is found in the coastal areas of northeastern Africa, the coastal areas of Pakistan (Sindh and Balochistan), and Northwest India (http://www.efloras.org/florataxon.aspx?flora_id=5&taxon_id=220013977, accessed on 11 September 2025). It is a perennial rhizomatous grass with a remarkable ability to withstand both salinity and drought [9,10,11,12]. It can survive up to 1000 mM NaCl during growth [9,10] and 500 mM NaCl during germination [11,12]. Seeds prefer a moderate temperature of 20/30 °C and a 12 h photoperiod for germination [11,12]. Mann et al. [13] performed de novo transcriptome profiling of *U. setulosa* leaves exposed to increasing salinity (300–500 mM NaCl), identifying differentially expressed genes and enriched pathways related to photosynthetic enzymes, mitogen-activated protein kinase (MAPK) signaling, transcription factors, transporter proteins, antioxidative enzymes, cell membrane proteins, and enzymes involved in the synthesis of compatible solutes, as well as other processes associated with salinity tolerance. This grass could be useful in coastal dune stabilization, landscaping/greenification of coastal areas, and as cattle/camel forage in saline areas [9,12,14,15]. Kumar et al. [16] reported the phytoremediation potential of *U*. *setulosa* in terms of reduction in soil salinity. The populations of *U. setulosa* in the coastal areas of Pakistan, especially along Hawke’s Bay Beach on the Karachi coast, are under increasing threat because of construction activities, overgrazing, and other disturbances [17,18]. Hussain et al. [18] classified *U. setulosa* as a threatened plant of the Karachi region. Low salinity tolerance during germination compared to growth level could be a bottleneck for its cultivation in the natural habitat for conservation and as a fodder crop on saline lands. Hence, there is a need to develop simple and low-cost methods to improve germination and salinity tolerance of this important fodder halophyte under saline conditions.

Seed priming is an easy, practical, cost-effective, and low-risk physio-chemical technique that improves seed germination and enhances seedling emergence and stress tolerance [19,20,21]. It is a simple hydration method in which seeds are subjected to limited imbibition to initiate the germination process without radicle emergence and then re-dried to their original weight [21,22]. Germination itself is a coordinated developmental cascade that requires the sequential activation of multiple tissues, including metabolic reactivation of the embryo, mobilization of reserves from storage tissues, and subsequent cell expansion leading to radicle emergence [22,23]. Controlled rehydration results in the activation of cellular processes such as the de novo synthesis of nucleic acids and proteins, ATP production, the accumulation of sterols and phospholipids, the activation of DNA repair and antioxidant mechanisms, and reserve mobilization [23], which altogether provide a head start to seeds for germination and subsequent seedling growth [23]. Under salinity stress, this coordination is often disrupted, as osmotic constraints delay embryo expansion and desynchronize reserve mobilization from growth demands, leading to delayed or arrested germination [1,19,24]. Seed priming is thought to enhance stress tolerance not by directly removing the stress factor but by improving the temporal coordination and efficiency of early germination processes, thereby reducing metabolic costs and preserving internal seed resources under adverse conditions [23,24,25]. A number of factors, such as seed type, vigor, priming duration, priming media, priming agent concentration, incubation period, and temperature, may influence the efficiency of seed priming [24,25]. However, in recent years, seed priming has emerged as a promising approach for stress management to protect plants against both biotic and abiotic stress conditions [26]. There are various types of seed priming, such as hydropriming, osmopriming, hormonal priming, halopriming, solid matrix priming, biopriming, and redox priming [27].

Seed priming with redox-active compounds, commonly referred to in the literature as “redox priming” [28,29,30,31], involves the controlled application of natural or synthetic agents such as hydrogen peroxide (H_2_O_2_; a prototypical reactive oxygen species), sodium nitroprusside (SNP; a nitric oxide donor), ascorbic acid (AsA; a major soluble antioxidant), and melatonin (a multifunctional antioxidant and signaling molecule) during seed imbibition [28,29,30,31]. Although the cellular redox environment is highly compartmentalized with distinct, semi-autonomous redox couples (e.g., ASC/DHA, GSH/GSSG, NAD(P)H/NAD(P)^+^, thioredoxins) operating in different organelles and at different developmental stages [30,31,32,33,34], the term “redox priming” is pragmatically used to describe pretreatments that induce a mild, transient perturbation of the cellular redox milieu [31]. These perturbations primarily involve the generation of controlled oxidative signals (e.g., via H_2_O_2_), nitric oxide release, or antioxidant supplementation, which collectively interact with major redox hubs, especially the ascorbate–glutathione (ASC-GSH) cycle and ROS-NO crosstalk pathways [31,32,33,34,35,36,37,38,39,40]. Such modulation helps maintain mitochondrial integrity/function, regulates cellular redox dynamics during early germination, and supports key developmental processes including seed germination, dormancy alleviation, seedling growth, and acquisition of stress tolerance [33,34]. Numerous studies have demonstrated that priming with these redox-active compounds significantly improves seed germination and early seedling vigor under various biotic and abiotic stresses. For instance, seed priming with H_2_O_2_ improved the tolerance of *Capsicum annuum* against chilling stress [35]. Ellouzi et al. [36] showed that seed priming with H_2_O_2_ alleviated salt effects by preventing ROS production and amplifying antioxidant defense in cauliflower seeds and seedlings. Seed priming with sodium nitroprusside improved salt tolerance in wheat by upregulating antioxidative defense mechanisms [37]. Sodium nitroprusside priming also improved seed germination and seedling growth in wheat under osmotic stress [38]. Seed priming with ascorbic acid improved drought resistance of wheat through an increase in endogenous AsA content, antioxidant potential, and osmotic adjustment [39]. Seed imbibition with melatonin improved salinity tolerance of basil plants by enhancing phenolic and flavonoid content [40]. However, similar information on halophyte seeds is generally scant. Hussain et al. [28] showed that priming with redox agents alleviated dormancy and enhanced salinity tolerance during germination and early seedling growth in the medicinally valuable halophyte *Zygophyllum simplex*. Similarly, ascorbic acid (AsA) priming improved germination under NaCl in *Suaeda fruticosa* and *Atriplex stocksii* [41]. Exogenous SNP and nitrate boosted salinity resilience at germination and seedling stages in *Suaeda salsa* [42]. Hydrogen peroxide (H_2_O_2_) pretreatment enhanced oxidative stress tolerance by upregulating antioxidant systems in several halophytes under salinity, including *Suaeda fruticosa* [43], *Cakile maritima* [44], and *Triticum aestivum* [45]. More recently, Hussain et al. [46] demonstrated that MT priming at varying concentrations significantly improved seed germination, seedling growth, and salinity tolerance in five halophytes: *Panicum antidotale*, *Portulaca oleracea*, *U. setulosa*, *Suaeda fruticosa*, and *Zygophyllum simplex*. These findings highlight the potential of redox-based priming to overcome early-life-stage bottlenecks in halophytes, yet comparative studies across C_4_ coastal grasses, particularly under controlled salinity gradients, are still scarce. This study examined the efficacy of different redox priming procedures on seed germination, seedling growth, and salinity tolerance of the halophyte grass *U. setulosa*. More specifically, answers to the following questions were explored: (1) Which redox agent(s) can enhance the germinability of *U. setulosa* seeds? (2) What are the optimal priming time(s) and concentration(s) to stimulate the germination of the test species? (3) Can redox priming improve salinity tolerance during seed germination? (4) Does redox priming improve seedling growth under both unstressed and stressed conditions?

## 2. Results

### 2.1. Effects of Redox Priming on Germination and Seedling Growth in Water

**Figure 1:**
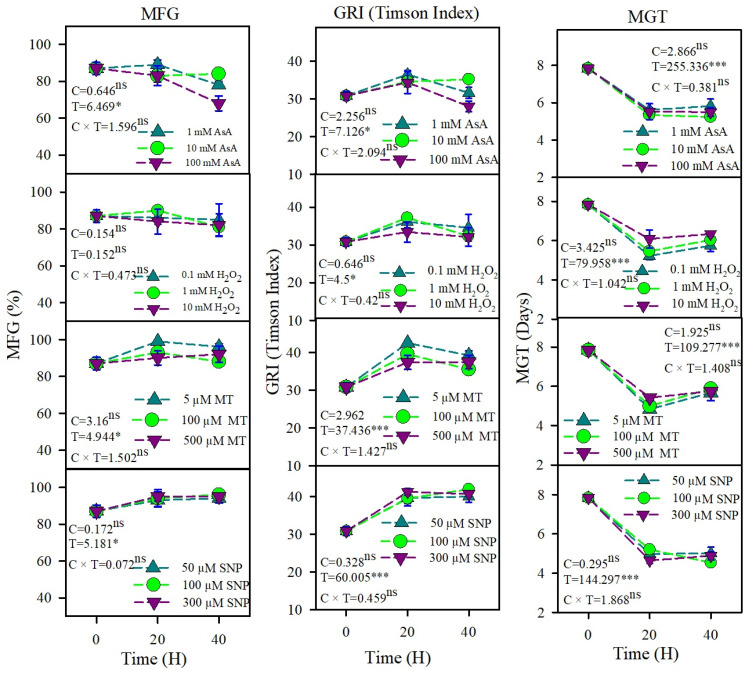
The effects of redox priming on mean final germination percentage (MFG), germination rate index (GRI), and mean germination time (MGT) of *U. setulosa* seeds in water. F-values of the two-way ANOVA for priming concentration (C), time (T), and their interaction (C × T) are given. * = *p* < 0.05, *** = *p* < 0.001, ns = *p* > 0.05 (non-significant).

Seeds of *U. setulosa* had 100% viability and showed ~90% germination in distilled water under optimal thermoperiod and photoperiod conditions. Analysis of variance indicated that priming durations (T) significantly affected mean final germination (MFG) for all agents except H_2_O_2_ and AsA, whereas priming agent concentrations (C) and the interaction of T and C did not. In contrast, the longest priming duration (40 h) combined with a high AsA concentration (100 mM) markedly reduced MFG compared to all other priming treatments and unprimed seeds. Among various redox agents, SNP and MT elicited the strongest and comparable enhancements in GRI and the most pronounced reductions in MGT, while H_2_O_2_ conferred moderate improvements, and AsA yielded the least enhancement relative to unprimed (0 h) controls (Figure 1). All priming treatments significantly reduced MGT compared to the unprimed control, with SNP and MT achieving the fastest germination (lowest MGT), followed by H_2_O_2_, while AsA showed the weakest effect (Figure 1).

**Figure 2:**
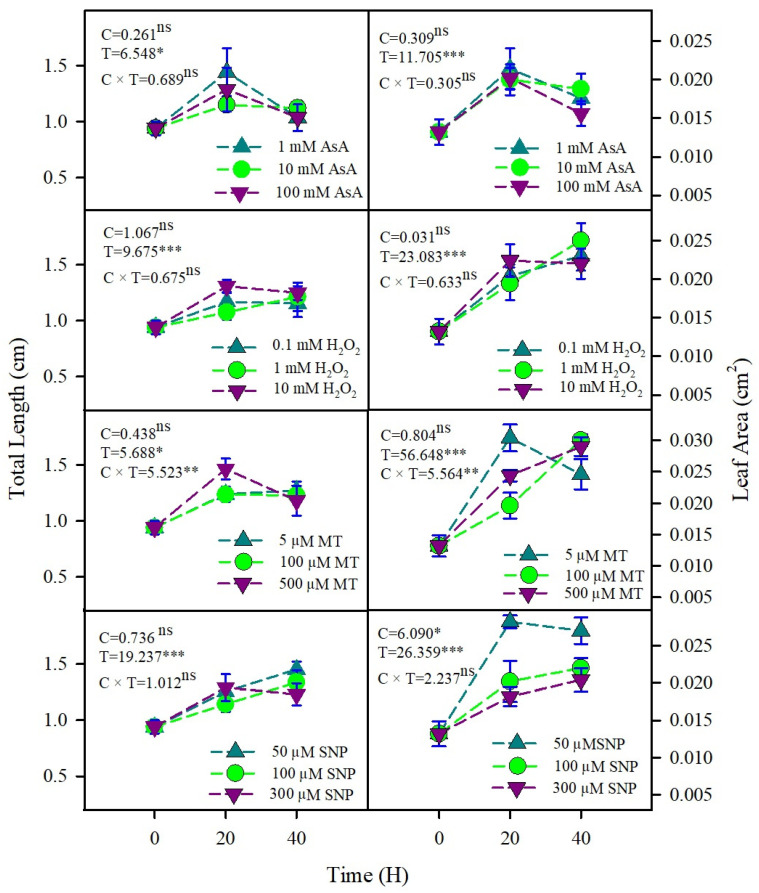
The effects of redox priming on total length and leaf area of *U. setulosa* seedlings in water. F-values of the two-way ANOVA for priming concentration (C), time (T), and their interaction (C × T) are given. * = *p* < 0.05, ** = *p* < 0.01, *** = *p* < 0.001, ns = *p* > 0.05 (non-significant).

Seedlings produced from primed seeds generally had significantly higher total length and leaf area in comparison with control seedlings (Figure 2). Similar to germination, priming duration but not concentration had a significant effect on seedling growth. Among the different priming treatments, MT, SNP, and H_2_O_2_ elicited the strongest enhancements in seedling growth (length and leaf area) traits, whereas AsA priming produced the weakest positive effects overall. Notably, extended priming durations with AsA failed to improve seedling growth relative to other priming agents and, in some cases, resulted in values comparable to or lower than those from unprimed seeds (Figure 2). Likewise, the chlorophyll content (Chl a and Chl b) of seedlings emerging from redox-primed seeds was significantly higher than that of seedlings from unprimed seeds. Among the priming treatments, MT and SNP showed the greatest effect on both Chl a and Chl b content compared to seedlings produced from unprimed seeds (Table 1).

### 2.2. Effects of Redox Priming on Germination and Seedling Growth Under Salinity

**Figure 3:**
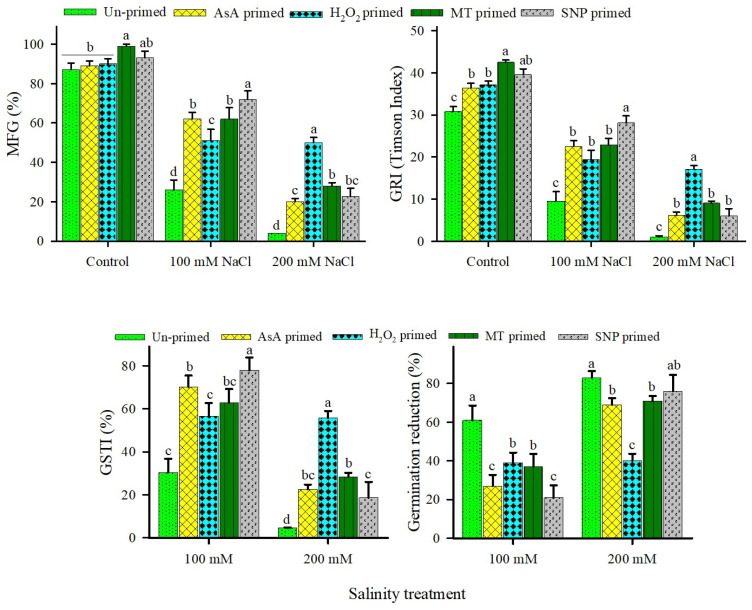
Effects of redox priming on mean final germination percentage (MFG), germination rate index (GRI), germination stress tolerance index (GSTI), and germination reduction (GR) percentage of *U. setulosa* seeds under different NaCl treatments. Data represents SE (n = 4). Bars with different letters indicate significant differences among treatments (*p* < 0.05; LSD test).

Increasing salinity led to significant reductions in germination of both unprimed and primed seeds (Figure 3). Only ~1% of unprimed seeds could germinate in 200 mM NaCl, with near-complete germination reduction (GR ~99%) and negligible germination stress tolerance index (GSTI; Figure 3). All redox priming treatments significantly alleviated this inhibition, markedly increasing MFG, GRI, and GSTI while reducing GR relative to unprimed controls (Figure 3). H_2_O_2_ priming conferred the greatest amelioration under severe salinity (200 mM NaCl), achieving the highest MFG, GRI, and GSTI alongside the lowest GR. At moderate salinity (100 mM NaCl), MT, SNP, and AsA elicited comparable improvements (Figure 3). Two-way ANOVA confirmed highly significant effects of salinity (S) and priming agent (PA) × S interactions on all germination parameters, including MFG, GRI, GSTI, and GR (green shading: *p* < 0.001; Table 2).

**Figure 4:**
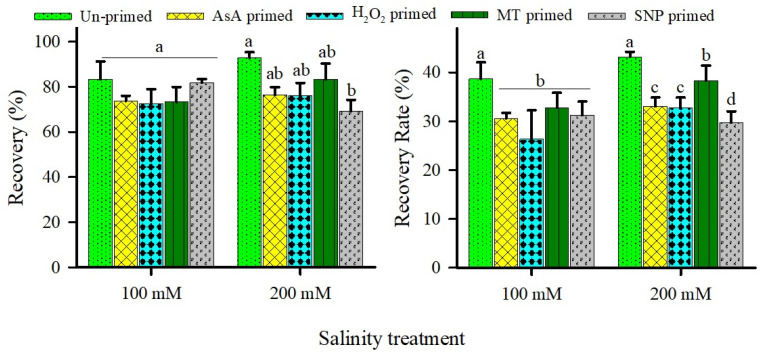
Effects of redox priming on recovery percentage and recovery rate index (RRI) of *U. setulosa* seeds under different NaCl treatments. Data represents SE (n = 4). Bars with different letters indicate significant differences among treatments (*p* < 0.05; LSD test).

Ungerminated primed and unprimed seeds from salinity treatments showed comparable recovery of germination when transferred to water (Figure 4). In general, ~90% recovery of germination of ungerminated seeds was observed after transferring from salinity to distilled water (Figure 4). Recovery and recovery rate index (RRI) followed similar trends, with bars sharing letters indicating non-significant differences (Figure 4).

**Figure 5:**
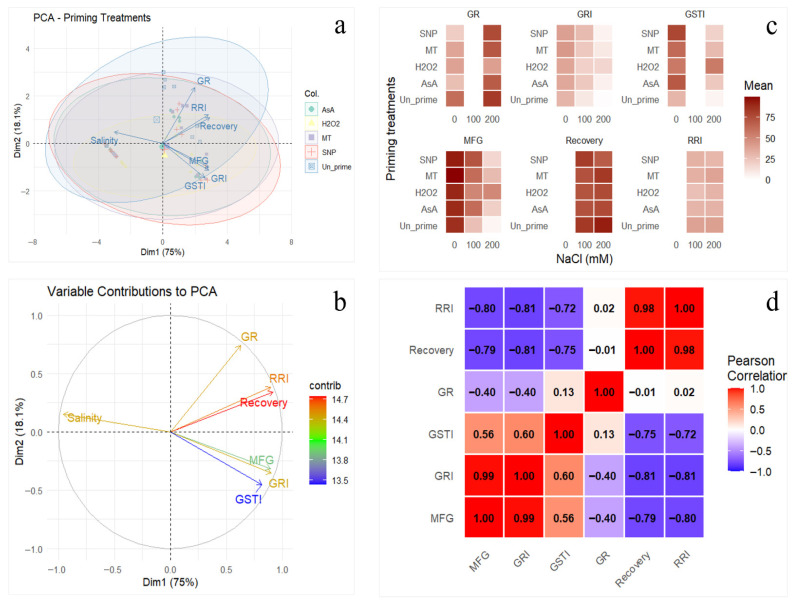
Multivariate analysis of seed germination performance under priming and NaCl stress. (**a**) PCA biplot showing separation of priming treatments (colored points) with 95% confidence ellipses. Trait loadings are shown as arrows. (**b**) Contribution (%) of each trait to PCA dimensions (color scale: blue = low, red = high). (**c**) Heatmap of mean trait values across treatments and NaCl levels (0, 200 mM). (**d**) Pearson correlation matrix of seedling traits (red = positive, blue = negative correlation). Traits: mean final germination (MFG), germination rate index (GRI), germination stress tolerance index (GSTI), and germination reduction (GR).

Principal component analysis (PCA) of germination traits confirmed clear separation of priming treatments (Figure 5a). PC1 (explaining the majority of variance) was strongly driven by MFG and GRI, while PC2 reflected germination reduction (GR) (Figure 5b). Heatmap visualization highlighted higher MFG and GRI across all priming agents, with MT and SNP clusters overlapping in high-performance regions (Figure 5c). Pearson correlation analysis showed strong positive correlations between MFG, GRI, and GSTI, contrasted by negative correlations with GR (Figure 5d).

**Figure 6:**
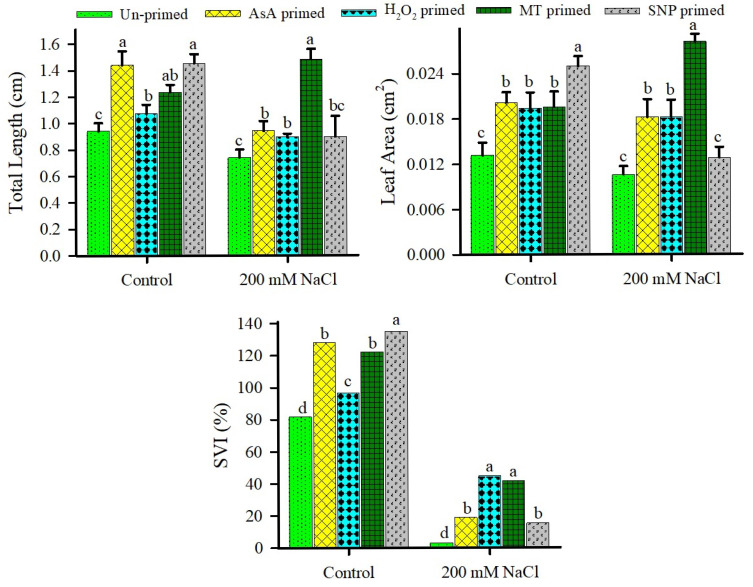
Effects of redox priming on total length, leaf area, and seedling vigor index (SVI) percentage of *U. setulosa* seedlings under different NaCl treatments. Data represents SE (n = 4). Bars with different letters indicate significant differences among treatments (*p* < 0.05; LSD test).

**Figure 7:**
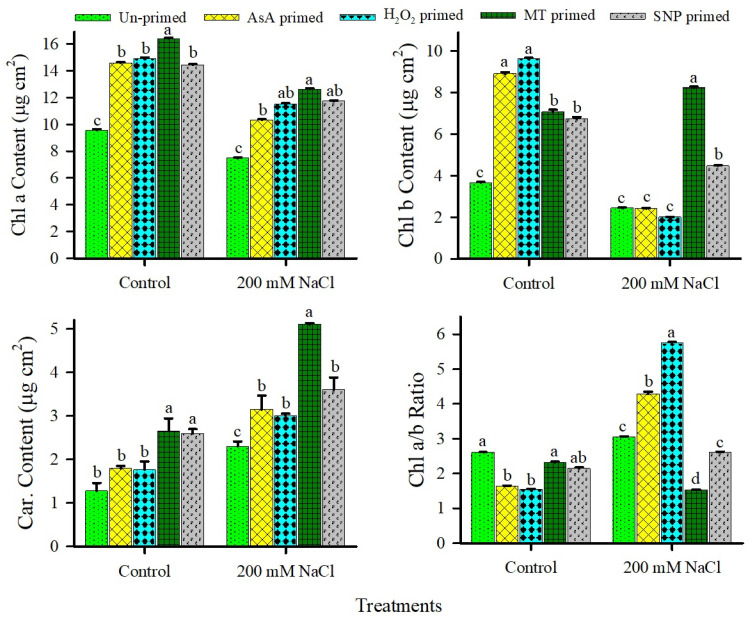
Effects of redox priming on chlorophyll (Chl a, Chl b), carotenoid (Car) content, and Chl a/b ratio of *U. setulosa* seedlings in the presence and absence of salinity. Data represents SE (n = 4). Bars with different letters indicate significant differences among treatments (*p* < 0.05; LSD test).

Contrary to expectations, total length and leaf area of the seedlings produced from unprimed seeds did not decrease with salinity (Figure 6). Seedling vigor index (SVI) in unprimed controls also showed a significant decline under 200 mM NaCl as compared to the control (Figure 6). Among priming agents, MT priming led to the highest enhanced total length, leaf area, and SVI under high salinity, conferring robust stress tolerance. AsA and H_2_O_2_ improved total length and SVI but not leaf area under saline conditions, while SNP priming boosted all three parameters under non-saline conditions but failed to mitigate salinity-induced constraints (Figure 6). Salinity induced minor declines in Chl a and Chl b contents of unprimed seedlings, with only minor improvement (not significant) effects on carotenoids (Car) and Chl a/b ratio (Figure 7). All priming agents elevated Chl a under both non-saline and saline regimes. MT and SNP further increased Chl b and maintained higher Car levels across conditions, resulting in stable Chl a/b ratios. In contrast, AsA and H_2_O_2_ enhanced Chl b and Car solely under non-saline conditions, with variable effects on Chl a/b under salinity (Figure 7).

**Figure 8:**
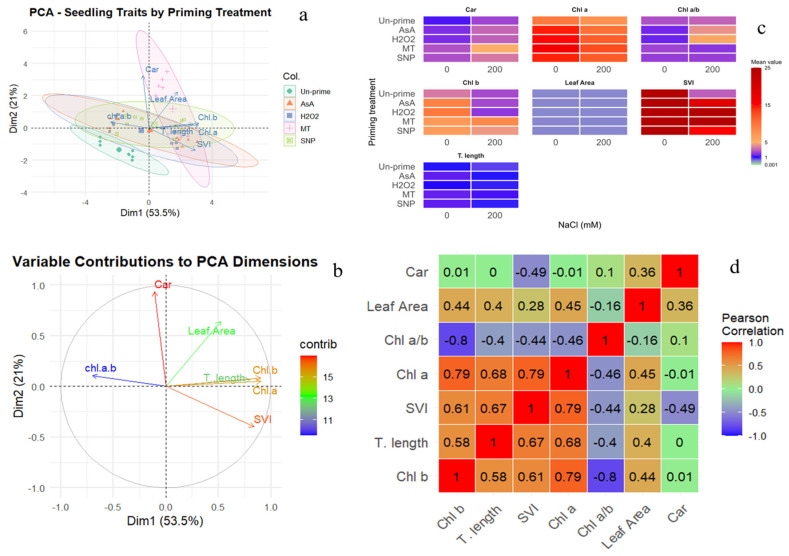
Multivariate analysis of seedling performance under priming and NaCl stress. (**a**) PCA biplot showing separation of priming treatments (colored points) with 95% confidence ellipses. Trait loadings are shown as arrows. (**b**) Contribution (%) of each trait to PCA dimensions (color scale: blue = low, red = high). (**c**) Heatmap of mean trait values across treatments and NaCl levels (0, 200 mM). (**d**) Pearson correlation matrix of seedling traits (red = positive, blue = negative correlation). Traits: T. length, leaf area, SVI, Chl a, Chl b, Car, and Chl a/b.

Multivariate analysis of seedling traits under salinity reinforced treatment separation (Figure 8a). PC1 was dominated by total length, leaf area, SVI, Chl a, and Chl b, whereas PC2 captured variability in carotenoids and Chl a/b (Figure 8b). Heatmap clustering highlighted MT-primed seedlings at both 0 and 200 mM NaCl, maintaining high trait values, particularly SVI, Chl a, Chl b, and Car, contrasting sharply with unprimed controls (Figure 8c). Correlation analysis confirmed strong positive relationships among SVI, length, and pigment parameters, suggesting that redox priming enhanced growth through coordinated physiological adjustments (Figure 8d).

## 3. Discussion

### 3.1. Effects of Redox Priming on Germination and Seedling Growth in Water

Seeds of *U. setulosa* lacked innate dormancy, as they showed ~90% MFG in distilled water at optimum photoperiod and thermoperiod. Gulzar et al. [11] and Shaikh et al. [12] have also reported similar results for seeds of *U. setulosa*. Seeds of other co-occurring C_4_ halophyte grasses, such as *Aeluropus lagopoides* [47], *Desmostachya bipinnata* [48], *Halopyrum mucronatum* [49], and *Sporobolus ioclados* [50], are also non-dormant and germinate maximally in water. Hence, lack of innate dormancy appears to be a common adaptation of halophyte grasses of the warm–subtropical region, which could be helpful in taking advantage of the brief monsoon period that mitigates soil salinity and drought in the natural habitat.

Priming of *U. setulosa* seeds with different redox compounds improved GRI but not MFG in water. However, all redox priming treatments significantly decreased MGT under non-saline conditions as compared to unprimed seeds. Likewise, priming with 1.5% KNO_3_ for 4 days did not significantly improve final germination but enhanced the germination index of *Quercus castaneifolia* seeds [51]. Similarly, *Brassica napus* seeds primed with different doses of AsA improved germination rate and uniformity, with significantly higher GRI and shorter MGT, without affecting the mean final germination percentage compared to unprimed seeds [52]. Seed priming with SA, GA, SNP, and ABA significantly improved the germination rate and shortened the MGT of two rapeseed cultivars [53]. Improvement in GRI can minimize the seedling formation time for the test species and thus would quickly maximize seedling establishment during the brief ‘moisture window’ after monsoon rains in warm–subtropical regions. In contrast, high AsA concentration combined with prolonged priming duration (100 mM, 40 h) reduced MFG. This response highlights the narrow hormetic window of AsA priming and suggests that excessive AsA exposure may disrupt, rather than optimize, early redox signaling. Although AsA is a central component of the cellular antioxidant system, its role during seed priming is highly context dependent. Under prolonged soaking or high concentrations, AsA is rapidly oxidized to dehydroascorbate (DHA). Importantly, DHA is chemically unstable and can further degrade into reactive carbonyl and acidic by-products, which may interfere with membrane integrity, enzyme activity, and hormone signaling during early germination stages, as emphasized in recent analyses [54]. Among various priming treatments, SNP (a nitric oxide donor) and MT led to the greatest improvement in the GRI and minimized the germination time of the test species. Nitric oxide (NO) and MT crosstalk have recently been established [55]. For instance, MT increases NO levels, probably via upregulation of nitrate reductase, and NO also causes MT biosynthesis through a cGMP-dependent pathway in plants [55,56]. MT reportedly modulates the expression of genes related to many germination-influencing phytohormones such as gibberellins, abscisic acid, and ethylene [56]. Roles of NO in the regulation of seed germination and dormancy have also been established [57,58,59]. Hence, crosstalk of MT and SNP might be responsible for improved GRI of *U. setulosa* seeds, which, however, needs to be confirmed in the future.

In this study, seedlings of *U. setulosa* after most priming treatments generally had higher growth and chlorophyll content as compared to those developed from unprimed seeds under non-saline conditions. SNP priming improved plant growth of *Triticum aestivum* seedlings in non-saline conditions compared to unprimed control seedlings [37], while H_2_O_2_ priming increased leaf biomass and expansion in *Brassica oleracea* [36]. However, such information on halophytes is limited and requires more studies. For instance, Hussain et al. [46] reported that MT priming at varying concentrations significantly improved total length, leaf area, and photosynthetic pigments in seedlings of *Panicum antidotale*, *Portulaca oleracea*, and *Zygophyllum* simplex under non-saline regimes. These findings underscore the growth-promoting potential of redox priming in unstressed environments, yet broader screening across C_4_ halophytic grasses is needed to fully elucidate underlying mechanisms and ecological implications.

### 3.2. Effects of Redox Priming on Germination and Seedling Growth Under Salinity

Salinity progressively reduced MFG, GRI, and GSTI while increasing germination reduction (GR) in *U. setulosa* seeds, consistent with earlier reports on this species [11,12]. However, unlike past studies that used seeds from the Hawke’s Bay coast of Karachi (Pakistan) with salinity tolerance of up to 500 mM NaCl, seeds of *U. setulosa* from the Gadani coast of Balochistan (Pakistan) in this study were comparatively less tolerant to salinity, and only ~1% of unprimed seeds could germinate in 200 mM NaCl. This difference could be attributed to intraspecific variations in germination and tolerance responses of the seeds from different provenances/populations due to differences in maternal/habitat environment [60,61,62].

All redox priming treatments significantly mitigated salinity-induced inhibition of germination in *U. setulosa*, markedly increasing MFG, GRI, and GSTI while substantially decreasing GR compared to unprimed controls (Figure 3). Under severe stress (200 mM NaCl), unprimed seeds exhibited near-total suppression (~1% MFG, ~99% GR), whereas H_2_O_2_ priming conferred the most robust tolerance, achieving the highest MFG, GRI, and GSTI alongside the lowest GR (Figure 3). Similarly, seed priming with H_2_O_2_ improved salinity tolerance during seed germination in *Capsicum annuum* [63], *Vicia faba* [64], and *Vigna unguiculata* [65]. Knowledge regarding the effects of redox priming on the salinity tolerance of halophyte seeds is limited. However, Hajihashemi et al. [66] reported that pretreatment of *Chenopodium quinoa* seeds with H_2_O_2_ led to faster and higher germination in saline conditions. Similarly, seed pretreatment with H_2_O_2_ improved salinity tolerance of germinating seeds of *Suaeda fruticosa* [43]. H_2_O_2_ is a reactive oxygen species (ROS) that regulates many plant processes, including seed germination and stress tolerance [67,68]. For instance, H_2_O_2_ regulated ABA catabolism and GA biosynthesis in *Arabidopsis* seeds for dormancy and germination [69]. Similarly, Rai-Kalal et al. [70] reported that H_2_O_2_ signaling regulated seed germination of primed seeds of *Triticum aestivum* under drought stress. H_2_O_2_ is also known to activate many tolerance responses in plants under various abiotic stresses, including salinity [71]. Similar underlying processes might be involved in improved salinity tolerance of *U. setulosa* seeds following H_2_O_2_ priming, which needs to be confirmed through detailed biochemical studies in the future.

Ungerminated primed and unprimed *U. setulosa* seeds from salinity treatments showed comparable high (~90%) recovery of germination when transferred to water. High germination recovery indicates that the germination failure resulted from enforced dormancy [72] due to osmotic but not ionic constraint of salinity [73]. Similarly, high recovery of germination was observed in other co-occurring halophyte grasses such as *Aeluropus lagopoides* [47] and *Desmostachya bipinnata* [48]. Hence, seeds of perennial C_4_ grasses of the warm–subtropical region appear to endure high soil salinity conditions, which could be hostile for seedling establishment, by entering a state of enforced dormancy and recovering their germination capacity as soon as soil salinity is diluted, such as after monsoon rains.

Unlike inhibitory effects on germination, salinity did not significantly decrease growth (length and leaf area) of the *U. setulosa* seedlings developed from unprimed seeds. Gulzar and Khan [14] also reported unaffected growth of *U. setulosa* mature plants under low to moderate salinity. Similarly, exposure to 200 mM NaCl did not decrease the growth of the co-occurring halophyte grass *Aeluropus lagopoides* [9]. Some C_4_ halophyte grasses, such as *Sporobolus virginicus* [74], *Stenotaphrum secundatum,* and *Paspalum vaginatum* [75], even grow better under saline conditions. Hence, C_4_ halophytic grasses appear to have better tolerance to salinity at the growth stage; however, information on the responses of the early seedlings of grasses is scant.

In this study, redox priming differentially modulated post-germinative growth under high salinity (200 mM NaCl). MT priming uniquely and significantly enhanced all growth parameters, achieving the highest total length, leaf area, and SVI, demonstrating superior salinity tolerance at the seedling stage (Figure 6). In contrast, AsA and H_2_O_2_ priming showed little improvement in total length, SVI, and leaf area, while SNP failed to confer any significant growth benefits under saline conditions, despite its efficacy in non-saline controls (Figure 6). MT is a pleiotropic regulator of plant development and stress adaptation, functioning as an antioxidant, growth promoter, and signaling molecule [76,77,78,79]. It is also emerging as a useful priming agent and reportedly improved salinity tolerance of many plants [40,78]. Seed priming with MT enhanced salinity tolerance of *Gossypium hirsutum* seedlings by regulating photosynthetic efficiency, enhancing ROS quenching, and coordinating with phytohormone signaling [79]. Likewise, MT priming improved salinity tolerance of *Phaseolus vulgaris* by ameliorating photosynthetic pigments, water relations, osmolyte accumulation, antioxidative defense mechanisms, and Na^+^ discrimination [80]. Such information on halophytes, especially C_4_ grasses, is scant. Recently, Hussain et al. [46] demonstrated that MT seed priming at varying concentrations significantly enhanced seedling growth and salinity tolerance in five halophyte species, including *Panicum antidotale*, *Urochloa setulosa*, *Portulaca oleracea*, *Suaeda fruticosa*, and *Zygophyllum simplex*. Similarly, Zhang et al. [81] reported that MT priming at 50 µmol L^−1^ markedly improved seedling vigor in *Suaeda corniculata* under NaCl stress by elevating antioxidant enzyme activities and reducing membrane lipid peroxidation. In the recretohalophyte *Limonium bicolor*, exogenous MT promoted salt secretion via salt glands through upregulation of ion transporter genes (e.g., SOS1, HKT1) and vesicle trafficking components [82]. A similar mode of action may be responsible for improved seedling performance of *U. setulosa* under saline conditions following MT priming, which, however, warrants detailed studies in the future.

Salinity elicited only minor, non-significant declines in Chl a and b contents of unprimed *U. setulosa* seedlings, with carotenoids (Car) and the Chl a/b ratio remaining largely unaffected (Figure 7). This pigment stability underscores the inherent salinity tolerance of *U. setulosa* at the early seedling stage, consistent with its C_4_ photosynthetic efficiency and halophytic adaptation, which likely prioritize photoprotective mechanisms and chloroplast integrity under moderate ionic stress [14,83]. All redox priming agents significantly elevated Chl a across both non-saline and saline conditions, suggesting a conserved role in chlorophyll biosynthesis or thylakoid stabilization during post-germinative development (Figure 7). MT and SNP further distinguished themselves by sustaining higher Chl b and Car levels under salinity, thereby preserving balanced Chl a/b as indicative of robust light-harvesting complex II (LHCII) assembly and photoprotective carotenoid pools (Figure 7). The superior pigment maintenance by MT and SNP under stress likely reflects their dual roles as antioxidants and signaling molecules. MT is known to scavenge ROS, upregulate carotenoid biosynthesis genes (e.g., PSY, LCY), and protect PSII reaction centers [77,78,82], while nitric oxide (from SNP) modulates chloroplast biogenesis and stress-responsive transcription factors (e.g., HY5, GLK1) to sustain pigment accumulation [37]. MT and SNP-mediated stabilization of the entire photosynthetic pigment profile under salinity likely contribute to sustained carbon assimilation and energy partitioning toward growth (as evidenced by enhanced SVI; Figure 6), reinforcing their potential as targeted priming agents for improving early-stage resilience in coastal C_4_ halophytes. Future studies should integrate chlorophyll fluorescence imaging, qPCR of pigment pathway genes, and metabolomic profiling to dissect the regulatory networks linking redox priming, pigment homeostasis, and salinity adaptation in *U. setulosa*.

**Figure 9:**
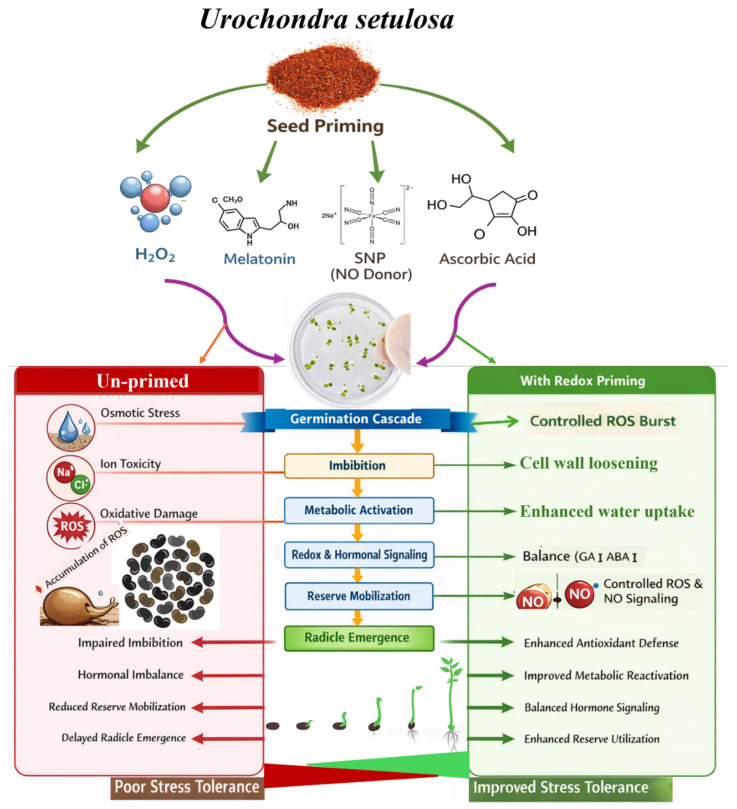
Conceptual model illustrates the effects of salinity stress and redox-based seed priming on the germination cascade and early seedling establishment in the coastal halophyte grass *Urochondra setulosa*.

Overall, salinity stress impairs the seed germination cascade in *U. setulosa* by delaying imbibition, slowing metabolic reactivation and reserve mobilization, and hindering radicle emergence and early seedling growth, as evidenced by reduced and slower germination kinetics, poorer seedling vigor, and lower chlorophyll content under saline conditions. Priming with redox-active compounds (H_2_O_2_, SNP, MT, and AsA) improves these outcomes by enhancing germination speed, final germination percentage, seedling growth, and pigment preservation, suggesting better coordination of the germination process and improved early establishment under stress. A conceptual model is proposed (Figure 9) that illustrates how salinity disrupts the cascade and how redox-based priming supports its progression in this halophyte. While specific biochemical or organ-level mechanisms were not investigated, the observed phenotypic improvements provide a foundation for this integrative framework.

## 4. Materials and Methods

### 4.1. Habitat, Seed Collection, and Initial Seed Quality Assessment

Mature seeds of *U. setulosa* were collected from a large population found in a dry-marsh pan near the Gadani Ship-Breaking Yard (latitude: 25°4′36.62″ N; longitude: 66°42′35.91″ E) in the Lasbela district, Balochistan, Pakistan. This area has warm–subtropical weather conditions with a mean annual precipitation of <350 mm and summer temperatures often exceeding 40 °C [84]. Seeds were randomly collected from a large number (>100) of plants of the entire population and taken to the laboratory. Seeds were separated from the inflorescence husk manually by scrubbing on a rubber car mat. Cleaned seeds were surface sterilized with 1% (*v*/*v*) sodium hypochlorite for 1 min, followed by thorough rinsing with distilled water and subsequent air-drying. Cleaned seeds were used in the experiments within a week of collection.

A preliminary germination test was carried out to determine initial germination attributes of the seed lot. Germination was carried out in tight-fitting plastic Petri dishes with 5 mL of the test solution and four replicates of 25 seeds each. Seeds were germinated in a programmed incubator having a 12 h light/12 h dark photoperiod and a 30/20 °C thermoperiod (i.e., the optimal germination environment for the test species [77]). Cool-white, fluorescent lamps (Philips; ~25 μmol m^−2^ s^−1^; 400–700 nm) were the light source. Seeds were considered to be germinated with the protrusion of radicles through the testa [24]. Seed germination was recorded on every alternate day for up to 20 days. The mean final germination percentage (MFG) and mean germination time (MGT) were determined according to the formulae given in Rasheed et al. [85]. The germination rate (GRI) was calculated by using a modified Timson Index of Germination Velocity: ∑G/t, where *G* is the percentage of seed germination at 2-day intervals, and *t* is the total germination period [86].

### 4.2. Experiment 1—Effects of Redox Priming on Germination and Seedling Growth in Water

This experiment determined the effective concentration and duration of each priming agent based on the mean final germination percentage (MFG) and germination rate (GRI). Stock solutions (100 mM) of H_2_O_2_, SNP, and AsA (all from Sigma-Aldrich, Singapore, Singapore) were prepared using distilled water. Melatonin (MT; Sigma-Aldrich) was first dissolved in 1 mL of 99% ethanol (to ensure complete dissolution, as MT is poorly soluble in water) and then diluted with distilled water to make a final volume of 100 mM stock solution. Working solutions of the desired concentrations were prepared by appropriate dilution of the stock solutions immediately before use. All light-sensitive reagents (H_2_O_2_, SNP, and MT) were stored in dark bottles and used immediately to prevent degradation.

Seed priming was carried out under continuous moderate laboratory light conditions at room temperature (25 ± 1 °C). Seeds (400 seeds per treatment) were separately imbibed in aqueous solutions of four redox compounds at different concentrations in tightly capped containers: (i) hydrogen peroxide (H_2_O_2_; 0.1, 1, and 10 mM), (ii) ascorbic acid (AsA; 1, 10, and 100 mM), (iii) sodium nitroprusside (SNP; 50, 100, and 300 µm), and (iv) melatonin (MT; 5, 100, and 500 µm) for 20 and 40 h. The selected concentrations and priming durations were chosen based on our previous studies on halophytes [28,46]. Once primed, the imbibed seeds were removed from the priming media, thoroughly rinsed with distilled water, and dried back at room conditions to their original moisture content on a fresh weight basis [22]. Untreated seeds served as the control. Seed germination of the primed and unprimed (control) seeds in water was carried out by following the method described above. During germination, the development of seedling growth was examined by following our previously established method [28,46]. Briefly, the five longest seedlings from each treatment were picked, and their total length and leaf area were measured from photographs using ImageJ software (version 1.54r). Chlorophyll of the seedling leaves was extracted in 1 mL of ethanol and estimated according to Lichtenthaler [87].

### 4.3. Experiment 2—Effects of Redox Priming on Germination and Seedling Growth Under Salinity

Based on experiment 1, seeds were primed with the most effective concentrations of redox-active priming agents for a specific time. Primed and unprimed (control) seeds were tested for salinity tolerance during germination and seedling growth stages. Seeds were exposed to different salinity levels (0, 100, and 200 mM NaCl) and their germination was examined for 20 days. The stress tolerance indices, mean final germination (MFG), germination rate index (GRI), germination stress tolerance index (GIST), and germination reduction percentage (GR) were calculated to evaluate the level of salt tolerance in both unprimed and primed *U. setulosa* seeds using the following equations [88]:

$$\mathrm{Mean}\;\mathrm{final}\;\mathrm{germination}\;\mathrm{percentage}\;(MFG)\;=\;\frac{Number\;of\;germinated\;seeds}{Total\;number\;of\;seeds}\;\times \;100$$

$$\mathrm{Germination}\;\mathrm{stress}\;\mathrm{tolerance}\;\mathrm{index}\;(GSTI)=\frac{MFG\;under\;stress}{MFG\;under\;control}\times 100$$

Germination reduction (GR)=MFG of control−MFG of stress

After 20 days, all ungerminated seeds from different NaCl treatments were thoroughly rinsed with distilled water and transferred to distilled water for another 20 days to study the recovery of germination from salinity stress. Recovery of germination was recorded on alternate days for 20 days. Final recovery percentage and recovery rate index (RRI) were determined according to Nisar et al. [84]. Growth of the seedlings developed during germination in various NaCl solutions (0 and 200 mM) was also examined by following the method detailed above. Total length, leaf area, and chlorophyll content of the seedlings were determined. Seedling vigor index (SVI) was obtained using the following equation given by Irik and Bikmaz [89]:

Seedling vigor index (SVI) = MFG (%) × mean seedling length

### 4.4. Statistical Analysis

Data were subjected to an analysis of variance (ANOVA) to determine if various priming concentrations and time affected germination and seedling attributes significantly. Germination/recovery percentage data were arcsine transformed for ANOVA. Homogeneity of variances was examined through Levine’s test. Mean values were compared with the help of the LSD test (*p* < 0.05). Statistical analyses were performed in SPSS software (version 22 for Windows). Pearson correlation analysis, heatmap visualization, and Pearson correlation matrix construction were conducted in RStudio (version 2025.05.1+513) to analyze the relationships among seed and seedling attributes and salt tolerance indices.

## 5. Conclusions

The seeds of *U. setulosa* showed ~90% MFG in distilled water under optimal conditions. Redox priming significantly accelerated germination rate (higher GRI and lower MGT) in non-saline conditions without altering final germination percentage. Under non-saline regimes, seedlings from primed seeds, particularly those treated with MT and SNP, displayed superior growth and elevated chlorophyll contents compared to unprimed controls. Increasing salinity severely inhibited germination of unprimed seeds under 200 mM NaCl. All redox priming agents mitigated this suppression by significantly enhancing germination attributes and stress tolerance, with H_2_O_2_ priming conferring the greatest tolerance. Ungerminated seeds from all treatments recovered ~90% germination, indicating enforced dormancy due to osmotic stress rather than irreversible damage. Remarkably, salinity did not impair post-germinative growth in unprimed seedlings. However, MT priming uniquely and significantly enhanced total length, leaf area, and seedling vigor index (SVI) under high salinity, outperforming AsA, H_2_O_2_, and SNP, which showed limited or no benefits under stress. MT and SNP priming resulted in higher chlorophyll and carotenoid levels, maintaining stable Chl a/b ratios under salinity, whereas AsA and H_2_O_2_ were less effective under stress. Multivariate analyses (PCA, heatmaps, and correlation matrices) confirmed MT as the most consistent priming agent for integrated performance across germination and seedling traits under salinity. These results demonstrate that redox priming, particularly with H_2_O_2_ for germination and MT for seedling vigor, offers a powerful, targeted strategy to enhance salinity tolerance during critical early life stages of *U. setulosa*. Such interventions hold significant promise for ecological restoration, coastal revegetation, and sustainable forage production in salt-affected arid environments.

## Figures and Tables

**Table 1:** Effects of different priming treatments on chlorophyll (a and b) contents of *U. setulosa* seedlings in water. Asterisk (*) over a primed value indicates a significant difference compared to unprimed values (*p* < 0.05; *t*-test).

Treatment	Concentrations	Time (h)	Chl a Content (µg cm^2^)	Chl b Content (µg cm^2^)
Unprimed (Control)	-	0	9.57 ± 0.08	3.66 ± 0.03
AsA	1 mM	20	13.89 ± 0.11 *	7.37 ± 0.06 *
		40	12.69 ± 0.06 *	7.17 ± 0.05 *
	10 mM	20	14.60 ± 0.04 *	8.91 ± 0.07 *
		40	10.27 ± 0.16 *	4.98 ± 0.02
	100 mM	20	11.46 ± 0.07 *	7.33 ± 0.05 *
		40	9.95 ± 0.05	4.40 ± 0.06
H_2_O_2_	0.1 mM	20	13.72 ± 0.06 *	6.62 ± 0.10 *
		40	12.13 ± 0.11 *	9.41 ± 0.08 *
	1 mM	20	14.93 ± 0.07 *	9.65 ± 0.02 *
		40	13.80 ± 0.15 *	11.46 ± 0.05 *
	10 mM	20	13.94 ± 0.06 *	10.21 ± 0.17 *
		40	12.19 ± 0.11 *	8.85 ± 0.10 *
MT	5 µM	20	16.39 ± 0.08 *	7.07 ± 0.10 *
		40	11.71 ± 0.09 *	5.17 ± 0.04 *
	100 µM	20	12.42 ± 0.13 *	5.25 ± 0.06 *
		40	12.38 ± 0.08 *	5.25 ± 0.09 *
	500 µM	20	12.03 ± 0.07 *	5.53 ± 0.08 *
		40	11.58 ± 0.04 *	4.52 ± 0.08
SNP	50 µM	20	14.19 ± 0.12 *	5.18 ± 0.05 *
		40	13.69 ± 0.15 *	6.53 ± 0.08 *
	100 µM	20	12.35 ± 0.20 *	4.60 ± 0.09
		40	13.93 ± 0.10 *	9.51 ± 0.05 *
	300 µM	20	14.45 ± 0.05 *	6.73 ± 0.10 *
		40	14.01 ± 0.06 *	6.14 ± 0.02 *

**Table 2:** Two-way ANOVA indicating the effects of redox priming agents (PA), salinity (S), and their interactions on various seed germination parameters of the test species. Green = *p* < 0.001, light green = *p* < 0.01, blue = *p* < 0.05, and red = *p* > 0.05.

Factors	MFG	GRI	GSTI	GR	Rec	RRI
Priming agents (PA)	0.18	0.04	0.30	0.38	0.34	0.04
Salinity (S)	0.00	0.00	0.28	0.01	0.55	0.22
PA × S	0.00	0.00	0.00	0.00	0.23	0.65

## Data Availability

The original findings and contributions presented in this study are included. Further inquiries can be directed to the corresponding author.
